# Back to life, back to reality: A multi-level dynamic network analysis of student mental health upon return to campus during the COVID-19 pandemic

**DOI:** 10.1007/s12144-022-03196-7

**Published:** 2022-05-12

**Authors:** Katharina Karnbach, Michał Witkowski, Omid V. Ebrahimi, Julian Burger

**Affiliations:** 1 PPLE College, Faculty of Law, University of Amsterdam, Amsterdam, the Netherlands; 2 Amsterdam University Medical Center, University of Amsterdam, Amsterdam, the Netherlands; 3 Department of Psychology, University of Oslo, Oslo, Norway; 4 Modum Bad Psychiatric Hospital and Research Center, Vikersund, Norway; 5 Department of Psychology, University of Amsterdam, Amsterdam, the Netherlands; 6 Centre for Urban Mental Health, University of Amsterdam, Amsterdam, the Netherlands; 7 Department of Psychiatry, Interdisciplinary Center Psychopathology and Emotion regulation (ICPE), University of Groningen, University Medical Center Groningen, Hanzeplein 1, 9713 GZ Groningen, the Netherlands

**Keywords:** Mental health, University students, COVID-19, Network analysis, Ecological momentary assessment

## Abstract

Lockdown measures during the COVID-19 pandemic resulted in drastic disruptions of university students’ everyday life and study mode, such as marked reductions in face-to-face teaching activities. Previous research on student mental health during the pandemic found that prolonged campus relocation had negative effects on students’ mental well-being. However, these studies focussed on the *initial* lockdown period, or periods of *active* lockdown measures. This longitudinal study collected 456 observations of 23 undergraduate students in the Netherlands using ecological momentary assessment data on mental health related items (anxiety, stress, social context) during the first two weeks of on-campus teaching after prolonged lockdown measures. Using multi-level dynamic network modelling, we analysed the temporal and contemporaneous interplay of students’ mental health factors following the return to campus in September 2021. On average, students reported low to medium scores on stress and anxiety both before and after the assessment period. Results of network analyses showed that students experienced social unease in relation to accumulating difficulties at university and vice versa. Furthermore, there were clusters of different states of social unease next to clusters of stress, anger, loss of control, and feeling upset. Lastly, we found beneficial effects of self-efficacy on experiencing social comfort in university. We discuss implications and concrete examples of interventions in universities, such as the promotion of self-efficacy, providing guidance in structuring study load, as well as help with stress management.

**Supplementary Information:**

The online version contains supplementary material available at 10.1007/s12144-022-03196-7.

University students mark a high-risk population for mental health problems, with substantially higher rates of mental disorder compared to those of the general population (Facundes & Ludermir, 2005; Gaspersz et al., 2012; Ibrahim et al., 2013; Stallman, 2010). Numerous studies have identified depressive and anxiety symptomatology as the most prevalent complaints (Ebert et al., 2019; Eisenberg et al., 2007; Holm-Hadulla & Koutsoukou-Argyraki, 2015; Storrie et al., 2010). Student mental health problems can adversely influence academic performance (Hysenbegasi et al., 2005; Storrie et al., 2010), and have been associated with a higher risk for substance abuse, such as alcohol consumption and cigarette smoking (Cranford et al., 2009; Tembo et al., 2017). Furthermore, students with mental health problems report less engagement on campus, poorer social relationships (Salzer, 2012), and worse perceived physical health (Hafen Jr et al., 2008).

## Student Mental Health and COVID-19

As Grubic et al. (2020) have argued, by increasing the mental burden in a population with heightened pre-existing stress levels, the ongoing COVID-19 pandemic and the concomitant lockdown measures since March 2020 have led to adverse mental outcomes (Ettman et al., 2020; Twenge & Joiner, 2020). Recent research has investigated the impact of lockdown and the closure of university on students. For example, Conrad et al. (2021) found that displacement from university-provided accommodation was associated with increased COVID-19 related grief, loneliness, and generalized anxiety symptoms, even when controlling for prior psychiatric diagnoses. Son et al. (2020) found that in the United States, 44% (86 out of 195 participants) reported suicidal thoughts in relation to the COVID-19 pandemic.

Conclusions drawn from the aforementioned studies may be limited due to their cross-sectional and retrospective designs, which may introduce recall biases. These limitations can be addressed by collecting *momentary* information, for example via *Ecological Momentary Assessment* (EMA; Myin-Germeys et al., 2018; Shiffman et al., 2008; Stone & Shiffman, 2018). EMA data are sampled repeatedly throughout the day via notifications from mobile devices, ensuring that data are entered in the real-life circumstances of the participants, which in turn increases ecological validity. Recently, new statistical methods have been developed that allow estimating within-person relationships from EMA data. These relationships can then be used to inform the construction of statistical network models (Burger et al., 2020, 2022; Epskamp, 2020; Epskamp, Borsboom, & Fried, 2018a; Epskamp, Waldorp, et al., 2018c), in which nodes represent (psychological) constructs (e.g., stress or anxiety), and connections between the nodes represent temporal (“from one time-point to the subsequent time-point”) or contemporaneous (“instantaneous”) relationships.

Some recent studies have analysed the network structure of EMA or daily diary data to better understand the complex interrelations connecting symptoms and mental health variables during the pandemic in the general population (Ebrahimi et al., 2021), and also specifically in university students (Fried et al., 2020). Copeland et al. (2021) found that nightly responses to an EMA survey exhibited a negative relationship between COVID-19 with mood and wellness behaviours throughout the semester. Fried et al. (2020) found and visualised a set of potential vicious cycles. Loneliness, mental health problems and concerns about COVID were positively related, which consequently predicted mental health outcomes, whereas spending time outdoors was linked to meaningful social activities. Most importantly, however, these EMA studies were able to follow and monitor the dynamic progression of students’ experiences under the circumstances of lockdown and university closure.

## The Present Study

In this study, we extend findings from previous EMA studies to a specific period in the pandemic that is currently under-investigated: The initial phase of returning to in-person teaching after a prolonged period of lockdown measures. The goal of this study was to investigate the impact of the return to campus on students’ mental health. During 1,5 years of lockdown and university closure, students had to adapt to a new reality, a new daily routine and new study methods (i.e. online classes, different exam formats). Especially drastic were the changes in students’ social context, due to the social contact restrictions enforced by the many governments during this period. In the Netherlands, for example, physical distancing measures included the closing of universities and schools, the closing of the hospitality sector and sports clubs, maintaining 1.5 m distance from others outside one’s own household by March 2020, a varying limit to the number of people allowed in one’s home and a temporary curfew, requesting people to stay inside between 20:30 and 04:30. Even when some institutions were allowed to open again by summer 2020, universities remained closed until September 2021.

A systematic review showed that during the lockdown, reported mean contact rates were reduced by 65–87% (Liu et al., 2021). Additionally, the authors found that students’ contact restrictions were especially severe, with a 100% elimination of school contacts, corresponding to school closure, as compared to working adults having experienced a 24–27% reduction in work contacts. Such drastic changes in social context can have adverse psychological effects, as prior research found the deterioration of social interaction, for instance resulting from lockdown measures, to have severe mental health implications in the context of COVID-19 (Godinić & Obrenovic, 2020; Kawohl & Nordt, 2020).

With universities reopening, students may be exposed to another strong change in their social environment, the effects of which are unpredictable. The extreme reduction in students’ amount of social contacts during university closure, and the potential harmful mental health effects they may have experienced in that time, highlight the importance of specifically investigating the social context aspect of students’ return to campus. By administering EMA assessments multiple times a day, this exploratory study attempts to examine the unprecedented effects of post-lockdown university life.

## Study Objectives

The aim of this study is two-fold. First, we aim to quantify the general frequency of self-reported mental health problems in students (anxiety, social anxiety, pandemic-related concerns, loneliness, stress) in the 2 weeks of the first on-campus semester. Specifically, we aim to capture potential changes over the study period when comparing the initial exposure to the new social context with the end of the study period. Second, we aim to investigate which variables (setting, frequency and type of social interaction) predict changes in mental health over the 2-week study period as well as dynamic relations among these potential predictor variables and mental health outcomes.

## Methods

### Procedure

The design of the present study is based on the study of Fried et al. (2020), who investigated student mental health during the COVID-19 lockdown, specifically focusing on the initial lockdown period in March 2020. We aligned our study design (procedure, scales, comparable sample characteristics, and sampling scheme) with the one of Fried and colleagues, as to provide direct comparison points between the two phases of the COVID-19 pandemic: On the one hand the context of the initial lockdown and university closure, and on the other hand the lifting of many measures for on-campus university teaching. One of the main strengths of Fried et al.’s (2020) study lie in their choice to collect EMA over 2 weeks, capturing experiences in the real-life contexts of students in the initial lockdown period. Furthermore, they administered entry and exit surveys, allowing them to compare changes on the dimension of validated scales before and after the EMA period, as well as changes that can be seen in the EMA time-series data. For this reason, we draw on a similar study design to address our research objectives.

Data collection took place between August 31, 2021, and October 4, 2021 and consisted of three parts: (1) a baseline assessment, (2) a 14-day EMA period, and (3) a post-assessment. In the first stage, participants filled in a 25-min Qualtrics survey. Two participants failed to meet the submission deadline and, therefore, were not included in the pre/post-measures analysis. After that, students proceeded to the second stage, namely 2 weeks of EMA. As the academic year of 2021/2022 began on the 6th of September, the EMA part started on Monday, September 6, and lasted until Sunday, September 19. Throughout this time, participants received notifications on their smartphones three times per day in fixed intervals, every 4 hours (at 10 a.m., 2 p.m. and 6 p.m.). Each assessment took about 3 minutes. If participants did not respond to an EMA assessment and did not complete the survey within the first 30 minutes, they received a reminder notification. The notification expired 60 minutes after the first notification. The EMA assessment was conducted via m-Path (https://m-path.io/; Mestdagh et al., 2022), a platform designed to collect momentary assessment data. In the last stage, students filled in a 15-min post-assessment Qualtrics survey.

### Participants

Participants were students of the undergraduate course *Politics, Psychology, Law and Economics* (PPLE) at the University of Amsterdam, who were recruited in the last two weeks of summer break via social networks and online student groups. To incentivise study participation, we organised a raffle for university merchandise. Moreover, participants were offered personalized feedback, which consisted of their own scores compared to the mean of the whole sample. Out of the 46 students that expressed interest in our study, 38 completed the baseline survey, 23 completed at least some of the EMA assessments (resulting in 456 EMA observations for all participants), and 22 completed the post-assessment survey. Out of the 20 participants who filled in both the pre- and post-assessment, 60% identified as female (*n* = 12) and 40% identified as male (*n* = 8). The mean age of the participants was 20.80 years (SD = 2.80, range = 18–32). We did not ask about the ethnicity and nationality of the participants. Most students were in their third year of university (*n* = 18), and the rest were in their second year (*n* = 2). Nineteen students were a part of the PPLE bachelor programme, and one was enrolled in the Psychology bachelor programme. Forty percent of the participants indicated that they were working next to their studies. Twenty-five percent of the students reported having suffered from mental health problems in the past or having taken psychiatric drugs. This is lower than what the findings of Ormel et al. (2015) on mental health in Dutch adolescents suggest, according to which the lifetime prevalence of mental disorders in late adolescence equals 45%. Moreover, Ormel and colleagues note that the lifetime prevalence tends to oscillate around 40% in other Western and industrialised countries as well. Differences may arise to the operationalisation of mental health problems. In our study, we merely asked participants directly for a history of mental health problems, which may have resulted in underreporting.

Teaching took place at the University of Amsterdam’s Roeterseiland Campus, hosting the faculties of law, economics and social sciences and providing lecture halls, study rooms, libraries, and a cultural center. Students usually live independently, as there is no on-campus accommodation provided for students. In this programme, students usually have four mandatory two-hour tutorials a week with about 15 classmates in regular classrooms, and four non-mandatory two-hour lectures a week in a bigger group of up to about 75 students. Students were allowed to join tutorials online if they had to quarantine in line with the regulations of the Dutch government (i.e., if they experienced symptoms, had recent contact to an infected person, or traveled to a country designated as high-risk country).

### Measures

We based our measures for all the assessment periods on those used by Fried et al. (2020). All measures can be found online https://osf.io/2qh98/.

#### Baseline and Follow-Up Assessment (Study Objective 1)

To address study objective 1, quantifying the potential changes in self-reported mental health problems in students, we conducted a baseline and follow-up assessment.

After being debriefed and asked for consent, participants responded to questions about their gender, age, year of study, Bachelor’s programme, employment status and prior mental health issues. Similar to Fried et al. (2020), we also asked additional questions to assess other psychological concepts. To this end, we used the same questionnaires that were adapted by Fried and colleagues, consisting of shortened versions of original scales to decrease participant burden (p. 4). The following scales were administered: 1) Previous-week depressive symptoms, anxiety symptoms, and stress was measured using the *Depression Anxiety Stress Scale* (DASS-21; Lovibond & Lovibond, 1996), consisting of 14 items per sub-scale (42 in total). Items are scored in terms of severity on a 4-point scale and summed per sub-scale. 2) Previous-month perceived stress was measured using the *Perceived Stress Scale* (PSS; Cohen & Williamson, 1998), consisting of 10 items measured on a 4-point scale. 3) General loneliness was assessed using five items from the eight-item revised *UCLA Loneliness Scale* (Russell et al., 1980) on a 4-point scale. 4) Frequency of social in-person activities was measured with a single item (“On average how many hours a day do you spend engaged in voluntary in-person social activity”). 5) Finally, self-efficacy was measured on using the General Self-Efficacy Scale (GSES; Zhang & Schwarzer, 1995), consisting of 10 items measured on a 4-point scale.

The post-assessment survey consisted of 77 items. Students were queried about depressive symptoms, anxiety symptoms, and stress, just as in the baseline survey. They were also asked about the impact of COVID-19 related stress and anxiety on their social lives and how well informed they felt by the University of Amsterdam and the Dutch government about the COVID-19 pandemic.

#### Ecological Momentary Assessment (Study Objective 2)

To address study objective 2, we collected EMA data on 20 questions assessing behaviour related to and experiences of mental health. The items were based on the prior study of Fried et al. (2020). In addition, to circumvent power limitations, and since the study specifically focuses on social factors, we opted for only including items that capture the experience of students in relation to changes in their social context, and not items that are specifically related to worries about COVID-19 itself.

**Fig. 1:**
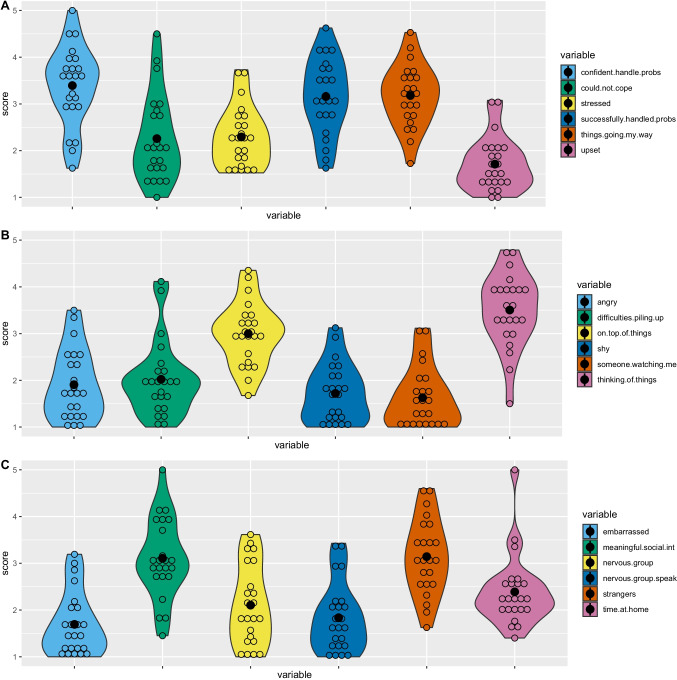
Distribution of EMA means per person and variable. Across-person means are indicated by the solid black circle, and person-wise means are indicated by empty circles

Furthermore, in contrast to Fried and colleagues, we used a time-window of 4 hours (instead of 3 hours). This change was made in order to account for the students’ lecture schedule and to avoid students missing the deadline of a notification because they were just beginning a class. All items were measured on a 5-point scale (1 = not at all, 2 = sometimes, 3 = often, 4 = very often, 5 = constantly” for items 1–10, and 1 = not at all, 2 = slightly, 3 = moderately, 4 = very, 5 = extremely for items 11–20). The mental health items were adapted from the Perceived Stress Scale (Cohen & Williamson, 1998) and the Social Anxiety Scale (Shevlin & Lewis, 1999). The full item list alongside means and standard deviations over the study period can be seen in Table 1, and overall variable means alongside person-wise means are visualised in Fig. 1. Then, the participants answered two questions regarding the amount of time they have spent on meaningful social interaction and time spent at home in the past 4 hours. Here, we used a multiple-choice format, with the possible answers including “0 min”, “1–15 min”, “15–60 min”, “1-2 h”, and “> 2 h”. For the analyses, we treated this variable as ordinal with five categories. The last two items were open-ended context questions (location and activity).

**Table 1:** Means and standard deviations of person-wise means on EMA variables

Item-label	Mean	SD
confident about ability to handle problems	3.39	0.84
could not cope with things I had to do	2.26	0.92
nervous and stressed	2.29	0.64
dealt successfully with problems	3.16	0.80
things were going my way	3.18	0.66
upset because of something unexpected	1.71	0.57
angered because of things outside of my control	1.91	0.74
difficulties are piling up	2.02	0.80
on top of things	3.00	0.67
taking time to get over shyness	1.72	0.62
hard to work when someone was watching	1.63	0.65
thinking about things I have to accomplish	3.50	0.78
embarrassed easily	1.69	0.68
time spent on meaningful social interaction	3.11	0.84
large groups make me nervous	2.10	0.85
nervous when speaking in front of group	1.84	0.73
easy to talk to strangers	3.14	0.80
time spent at home	2.39	0.76

### Statistical Analyses

To address the first study objective (i.e., quantifying frequency of and changes in mental health problems), we calculated descriptive statistics (mean and standard deviations) of the EMA variables and conducted paired-sample t-tests between the pre- and post-assessment.

To address the second study objective, we used multi-level vector autoregressive (mlVAR) models to estimate time series networks from the EMA data (Bringmann et al., 2013; Burger et al., 2022; Epskamp, Waldorp, et al., 2018c). This model can be used to calculate (fixed) *within-person relationships*, which can subsequently inform the construction of two types of networks: First, a directed *temporal* network, which represents average within-person relationships over time, commonly using a lag-1 approach (i.e., predicting current responses to a variable from responses on all other variables during the previous assessment). In other terms, temporal networks in this context allow for inferences such as “*Reporting increases in variable A is followed by increases/decreases in variable B four hours later*”. Second, an undirected *contemporaneous* network, which represents average within-person relationships occurring within the same assessment (i.e., using the residuals of the temporal prediction). In other terms, contemporaneous networks in this context allow for inferences such as “*Reporting increases in variable A is associated with increases/decreases in variable B within the same assessment*”. The multi-level approach entails modelling these temporal and contemporaneous relationships as fixed effects (i.e., effects of an “average” person), where each effect is established as a normal distribution of all individual (“random”) effects.

To conduct the analyses, we used the R-package *mlVAR* (Epskamp et al., 2017; version 0.4.4 on the 14th of December 2021). Networks were visualized using the *qrgaph* package in R (Epskamp et al., 2012; version 1.6.9 on the 14th of December). Prior to estimating networks, we conducted several pre-processing steps that are common for this type of time series data, including the removal of linear time trends and excluding participants with less than 20 observations (Epskamp, van Borkulo, et al., 2018b; Jordan et al., 2020). We include the R-script in an online supplementary folder https://osf.io/2qh98/.

## Results

### Frequency of Self-Reported Mental Health Problems

Figure 1 shows violin plots with person-wise means and distributions for all EMA variables. In addition, we list a numeric summary (means and standard deviations) of the EMA variables in Table 1. Variable means ranged from 1.63 (“*It was hard for me to work when someone was watching me.*”) to 3.60 (“*How often have you found yourself thinking about things that you have to accomplish?*”), indicating that in general, students did not experience high levels of stress and anxiety in the assessment period.

### Changes in Self-Reported Mental Health Problems

**Fig. 2:**
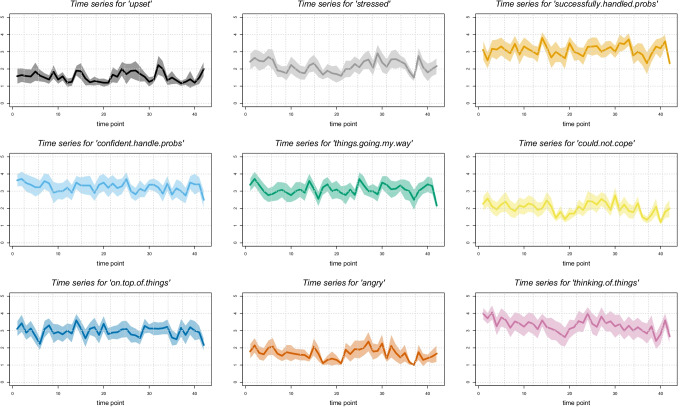

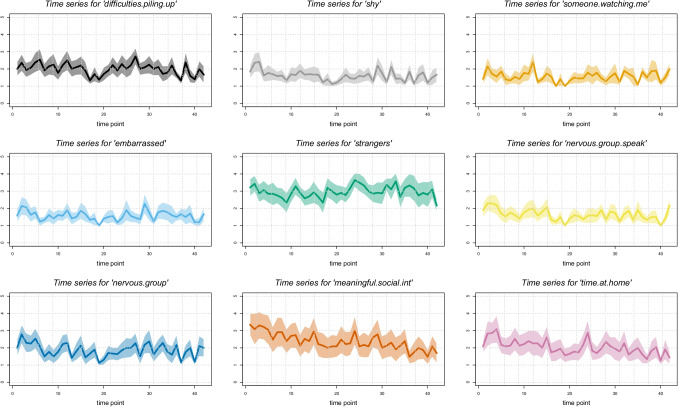
**a** Time series of all EMA variables. The solid line represents the mean across individuals for each time point, the shaded area indicates the 95% confidence interval of the across-person mean. **b** Time series of all EMA variables. The solid line represents the mean across individuals for each time point, the shaded area indicates the 95% confidence interval of the across-person mean

Figures 2a–b visualise the results of the time series prior to pre-processing (i.e., before removing linear trends and scaling), indicating the across-individual average changes of all investigated variables per day. Overall, the time series were relatively stable across the assessment period. Some significant, albeit weak, trends were found: On average, there was a decrease in participants’ feeling that they *could not cope with things they had to do* (*b* = −.009), that *difficulties were piling* up (*b* = −.008) and in *thinking of things they had to do* (*b* = −.015). Further decreases were observed for participants’ *confidence about their ability to handle problems* (*b* = −.008), *taking time to get over shyness*(*b* = −.008), *feeling nervous when speaking in front of a group* (*b* = −.010), *time spent at home* (*b* = −.02), and *time spent on meaningful social interactions* (*b* = −.03). It is important to note, however, that these effects were small according to effect-size conventions (ranging from *ƒ*^2^ = .00006 for *confidence about ability to handle problems* to *ƒ*^2^ = .0009 for *time spent on meaningful social interaction*), indicating that changes over time may not be of relevance in this context. The remaining variables did not show any significant changes.

The pre- and post-assessment showed that scores remained in their respective initial severity labels (Lovibond & Lovibond, 1996), with the *depression* (*M*_*t1*_ = 12.37, *SD*_*t1*_ = 5.30; *M*_*t2*_ = 12.58, *SD*_*t2*_ = 4.96) and *stress* (*M*_*t1*_ = 14.95, *SD*_*t1*_ = 3.60; *M*_*t2*_ = 14.20, *SD*_*t2*_ = 4.00) scores remaining in the “mild” and *anxiety* scores (*M*_*t1*_ = 12.47, *SD*_*t1*_ = 4.00; *M*_*t2*_ = 12.21, *SD*_*t2*_ = 4.50) remaining in the “moderate” categories. Paired sample t-tests revealed that none of these scales showed significant differences comparing pre- with post assessment; *t*(18) = 1.03, *p* = 0.316 for *stress*, *t*(18) = 0.40, *p* = 0.697 for *anxiety*, *t*(18) = −0.26, *p* = 0.796 for *depression*, and *t*(20) = 1.51, *p* = 0.147 for *loneliness*.

### Network Analysis of EMA Variables

**Fig. 3:**
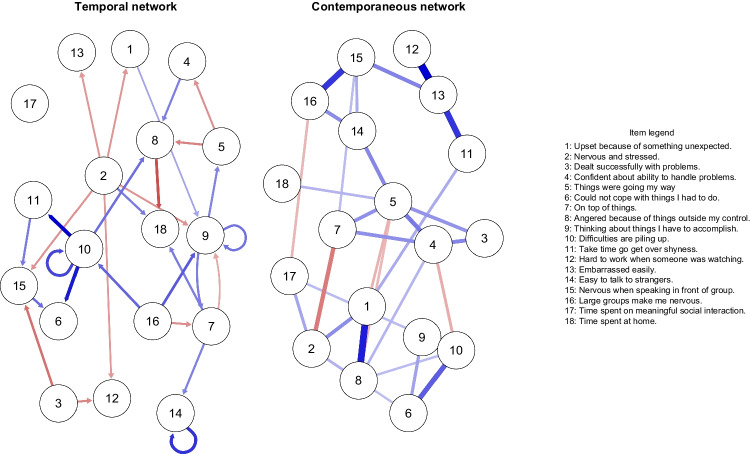
Fixed effects temporal and contemporaneous network for the EMA data. The temporal network (left) indicates relationships between variables across the assessment points, whereas the contemporaneous network (right) indicates relationships occurring within the same window of measurement. Blude edges represent positive relationships, red edges represent negative relationship. Edges in the temporal network range from *r* = −.22 (8 ➔ 18) to *r* = .31 (10 ➔ 11), and in the contemporaneous network from *r* = −.20 (2–7) to *r* = .39 (12–13)

Figure 3 shows the temporal (left) and contemporaneous (right) network, displaying within-person patterns of interplay. Specifically, they provide insight into how the investigated variables predict each other from one measurement window to the next (temporal) and co-occur within the same measurement window (contemporaneous). We include both corresponding adjacency matrices in the online appendix.

#### Temporal Network

The temporal network (Fig. 3, left panel) shows the average within-person connections between nodes from one measurement to the next. In this network, edges are *directed* as they indicate a particular temporal sequence. For example, a positive (solid-blue) arrow from variable A to variable B shows that A is positively associated with B *at the subsequent assessment*. In the time series literature, temporal effects are sometimes referred to as encoding so-called “Granger causality” (Granger, 1969), however, it needs to be noted that these effects are merely temporal predictive effects, and that inferring “true” causality is indeed a more complex endeavour (Dablander, 2020; Pearl, 2011).

The results of the temporal network indicate that students found it difficult to handle social situations and their study load as a consequence of experiencing accumulating difficulties. This can be seen in a positive effect of *feeling that difficulties were piling up* on *taking time to get over shyness* (*r* = .31), on *could not cope with things* (*r* = .28), and on *feeling angered because of things outside their control* (*r* = .18) at the subsequent assessment. The *feeling of difficulties piling* up was also predictive of higher within-person levels of this same feeling at the next measurement time (*r* = .23), indicating that students found it generally difficult to decrease burden resulting from these tasks by themselves. Furthermore, students may have experienced stress due to reviewing previous socially uncomfortable situations in detail, a phenomenon referred to as post-event processing (Clark & Wells, 1995). More specifically, post-event processing concerns the reconstruction of social events ensuing their occurence, characterized by ruminative process which facilicatates the reconstruction of the event in a negative light, further making the person more prone of anticipating future events to be negative. This can be seen in the positive temporal effect of *feeling nervous in large groups* on *ruminating about future accomplishments* (*r* = .21), as well as the negative effect on *feeling on top of things* (*r* = −.14). In line with the detrimental effects of social unease, we found that *feeling nervous when speaking in front of a group* predicted an increased *feeling of not being able to cope with the things* (*r* = .18) at the subsequent assessment. Interestingly, different contextual settings had positive effects on the students’ well-being. For example, *spending time at home* was predictive of feeling less *angry about things outside one’s control* (*r* = −.22). Lastly, our results align with previous research indicating that students’ self-efficacy is playing an important role for feelings of social unease (Thomasson & Psouni, 2010), as their experience of *successfully handling problems* was associated with them being more socially resilient in subsequent assessments, in particular feeling less *nervous when speaking in front of a group* (*r* = −.18), and finding it less *hard to work when someone is watching* (*r* = −.15).

#### Contemporaneous Network

The contemporaneous network (Fig. 3, right panel) shows the average within-person relationships among the investigated nodes taking place within the same window of measurement. Furthermore, as effects in the temporal network are dependent on the chosen time-lag (in this case 4 h), some of the processes that occur on a faster time scale might be captured in the contemporaneous network rather than the temporal network. Edges in the contemporaneous network are *undirected*, as they do not correspond to a specific time sequence.

In the contemporaneous network, we identified a cluster of variables relating to social unease: Increased *feelings of getting embarrassed easily* were associated with *taking more time to get over shyness* (*r* = .31), *feeling more nervous when speaking in front of a group* (*r* = .19), and finding it *harder to work when someone was watching* (*r* = .39). An increased feeling of *being nervous in large groups* was further associated with *feeling more nervous when speaking in front of a group* (*r* = .31). Similar to findings in the temporal network that, the contemporaneous network also supports detrimental effects of experiencing accumulating difficulties. In particular, we observed a positive relationship between *feeling that difficulties were piling up* with *thinking of things one has to accomplish* (*r* = .24), as well as with *feeling unable to cope with the things one has to do* (*r* = .25). Furthermore, the beneficial effects of self-efficacy as found in the temporal network could also be identified in the contemporaneous network. Here, increased feelings of *confidence about one’s ability to handle problems* were associated with higher levels of *feeling that things were going one’s way* (*r* = .21), and *having dealt successfully with problems* (*r* = .18). Conversely, we found that negative aspects of students’ experience, such as stress, anger, loss of control, and feeling upset tended to co-occur, potentially indicating a negative spiral of detrimental effects. For example, we found that *feeling nervous and stressed* was inversely related to *feeling on top of things* (*r* = −.20), and *feeling anger because of things outside of one’s control* was positively related to *being upset because of something unexpected* (*r* = .34).

## Discussion

Our findings suggest that, overall, undergraduate students experienced low to medium levels of anxiety, stress, depression, and loneliness immediately before, during, and after the initial return to campus in September 2021. Their scores on all measures did not indicate particular struggles and remained quite stable throughout the data collection period. There were no significant changes between measurement scores in the very beginning of the semester and the last measurement time after 2 weeks.

In sum, psychological complaints were mild to moderate and remained stable throughout the study period. We see at least five potential explanations for this: First, students may have experienced the return to campus indeed as a positive event, in turn limiting the extent to which psychological complaints were experienced. Second, students may have already started to become socially more active prior to the event of campus relocation, meaning that potential stressful effects of this event already occured prior to the actual assessment. Third, the study period may have been not long enough to capture actual changes in these variables. Fourth, as we discuss later on, we have primarily focused on the aspect of social interaction. It is possible that other factors, such as fear of contagion or preliminary protection via vaccines, may have led to stable scores. Fifth, mental health complaints may be underestimated because participants who signed up for this study may overrepresent individuals who are not that impacted by the pandemic, as participation requires daily assessments.

Additionally, our study provides exploratory insight into the importance of experienced social and psychological dynamics in university students: Overall, states of social unease (e.g., feeling nervous in large groups) predicted difficulties functioning in university contexts over time and vice-versa, potentially indicating the presence of post-event processing. Furthermore, different contexts and experiences of social unease reinforced each other, as did psychological experiences of stress, anger, loss of control, and feeling upset. Conversely, we identified potential mental health promoting relationships between variables, such as the positive effects of variables relating to self-efficacy on social functioning.

These findings may have implications for fostering a supportive environment for university students, specifically in an extraordinary context such as returning to campus: To intervene on the negative cycles of stress and loss of control, students may benefit from the promotion of stronger guidance regarding time management and balancing their study load, as well as stress management. Furthermore, specific attention should be directed to promoting students’ self-efficacy, which according to our findings could have a beneficial effect on social functioning. Feeling socially at ease, in turn, could therefore make it easier for students to engage with their study load and university tasks, as indicated by our findings. In the context of this study, positive effects of self-efficacy specifically refer to the increased resilience that follows the experience of successfully handling one’s problems. Individuals who felt they were on top of things also exhibited a stronger sense of resilience towards other problems. This importance of promoting self-efficacy in students in the context of COVID-19 has also been found in prior publications (Blanco et al., 2020).

Concrete suggestions for implementing self-efficacy interventions are provided by Margolis and McCabe (2006). Specifically in the context of returning to in-person teaching, attention should be paid to administering *moderately-difficult* assignments, acknowledging that students may lack some skills they would have acquired in offline teaching, such as giving in-person presentations, engaging in debates and group discussions. Furthermore, it may generally be beneficial to address these differences in learning offline versus online, and focus specifically on teaching learning strategies that students have missed out on previously. General aspects of increasing self-efficacy according to Margolis and McCabe (e.g., encouraging, providing frequent and focused feedback, capitalising on student” interests) may also be important in this context. In addition, another line of research describes that the self-efficacy of teachers may also play an important role in the students’ experience (Hoy, 2004), indicating that stimulating exchange across teaching staff, and support from programme managers may also be fruitful interventions.

### Strengths of the Study

To our knowledge, at the time of writing this paper, the present study is the first one to look at student adaptation to returning to campus after an extended period of lockdown and an extended period of online classes. The design employed in this research takes advantage of intensive longitudinal data, which circumvents many of the issues common to cross-sectional design. EMA methodology avoids recall biases encountered in retrospective data collection by asking participants to report how they feel *here* and *now*, rather than having them reflect upon a longer period of experience that might be less clear and tangible. Together with questions that aimed to record contextual information, our design allowed us to examine how the mental variables were shaped *in the moment* and how a specific context might have influenced the experience.

Second, the items used in the EMA part cover a broad range of mental health related aspects, including psychological states, social and contextual variables, as well as behavioural indicators. Our study is therefore targeting a broad understanding of mental health that is not restricted to affective states, and therefore contributes to the conceptualisation of mental health as systems of biological, social, and psychological components.

Another strength of the present paper lies in its analytic approach. Here, we used multi-level dynamic network analyses to model the interplay between mental health variables. First, we used a multi-level approach which allows for more powerful estimation. Second, the resulting models reveal dynamic *within-person* relationships that can be used to generate hypotheses on the daily dynamics in the lives of individuals. Longitudinal within-person analyses are better suited for dynamic interpretation compared to between-subject analyses, because the latter merely focus on one cross-section of time, and therefore rely on the very strong assumption of *ergodicity* (Molenaar, 2004) when making within-person claims.

### Limitations of the Study

It must be acknowledged that the present study was subject to limitations. First, our sample exclusively consisted of undergraduate students from one study programme and might thus not be representative of university students in general. Additionally, there was potential for selection bias since people who were struggling might not have signed up to participate or might have dropped out of the study during data collection. The opposite might have happened as well–it could be argued that those who have problems decided to join the study as they are more preoccupied with thinking of and finding out about these problems.

Second, it is unclear when the stressor we are interested in (returning to campus) affected people, or even if the event was experienced as stressful or indeed positive. The event might have already affected students prior to the data collection, e.g., in the weeks directly preceding students’ return to campus, which would also explain the relative stability of symptoms over the assessment period. Additionally, the role of students’ fear of Covid-19 has not been investigated in this study, however, this might have shaped students’ social context experiences on campus. Furthermore, we have not collected information on the vaccination status of the participants, which could explain some of the experience in social comfort.

Third, it is currently not possible to determine precise power requirements for multi-level VAR studies. Preliminary power simulations for fully idiographic network analyses indicate that more time points than the ones used here could be required (Mansueto et al., 2022), however, one of the advantages of multi-level network estimation is that fixed within-person effects are informed by other individuals in the sample, which facilitates estimation and is therefore generally more powerful than fully personalized routines (Burger et al., 2022; Epskamp, Waldorp, et al., 2018c). Nevertheless, future studies should aim at recruiting more individuals and collect longer time series. In this study, we decided to collect data only for a period of 2 weeks, because it can be assumed that longer time periods may threaten the assumption of *stationarity* in vector autoregressive modelling, which posits that models are assumed to be time invariant (do not change over time).

## Conclusions

Students reported relatively low to moderate medium mental health struggles that were stable across the initial two-week period upon return to campus during the COVID-19 pandemic. The network analyses revealed temporal relationships between social unease and problems at university, contemporaneous clusters of different states of social unease, as well as positive effects of self-efficacy on students’ university functioning and social comfort. These findings could give rise to interventions such as promoting self-efficacy in students, as well as providing guidance in structuring their study load and stress-regulation strategies.

## Supplementary Information

ESM 1 (CSV 6 kb)

ESM 2 (CSV 5 kb)

ESM 3 (R 9 kb)
